# Integrative Analysis of Methylation and Transcriptome Identified Epigenetically Regulated lncRNAs With Prognostic Relevance for Thyroid Cancer

**DOI:** 10.3389/fbioe.2019.00439

**Published:** 2020-01-09

**Authors:** Qiuying Li, Peng Wang, Chuanhui Sun, Chao Wang, Yanan Sun

**Affiliations:** ^1^Department of Otorhinolaryngology, Head and Neck Surgery, The Second Affiliated Hospital, Harbin Medical University, Harbin, China; ^2^Department of Otorhinolaryngology, The First Affiliated Hospital, Guizhou University of Traditional Chinese Medicine, Guiyang, China

**Keywords:** thyroid cancer, long non-coding RNAs, DNA methylation, biomarkers, prognosis

## Abstract

Emerging evidence has shown that epigenetic changes in DNA methylation, an important regulator of long non-coding RNA (lncRNA) expression, can disturb the expression patterns of lncRNAs and contribute to carcinogenesis. However, knowledge about crosstalk effects between DNA methylation and lncRNA regulation in thyroid cancer (THCA) remain largely unknown. In this study, we performed an integrated analysis of methylation and the transcriptome and identified 483 epigenetically regulated lncRNAs (EpilncRNAs) associated with the development and progression of THCA. These EpilncRNAs can be divided into two categories based on their methylation and expression patterns: 228 HyperLncRNAs and 255 HypoLncRNAs. Then, we identified a methylation-driven 5-lncRNA-based signature (EpiLncPM) to improve prognosis prediction using the random survival forest and multivariate Cox analysis, which were then validated using the training dataset [Hazard ratio (HR) = 50.097, 95% confidence interval (CI): 10.231-245.312, *p* < 0.001] and testing dataset (HR = 4.395, 95% CI: 0.981-19.686, *p* = 0.053). Multivariate analysis suggested that the EpiLncPM is an independent prognostic factor. By performing a functional enrichment analysis of GO and KEGG for mRNAs co-expressed with the EpiLncPM, we found that the EpiLncPM was involved in immune and inflammatory-related biological processes. Finally, *in situ* hybridization analysis in 119 papillary thyroid carcinoma (PTC) tissues and paired adjacent normal tissues revealed that selected candidate lncRNA *AC110011* has significantly higher expression of PTC compared to adjacent non-neoplastic tissues, and was closely related to the tumor size, lymph node metastasis, and extrathyroidal extension. In summary, our study characterized the crosstalk between DNA methylation and lncRNA, and provided novel biomarkers for the prognosis of THCA.

## Introduction

Thyroid cancer (THCA) constitutes one of the most frequently diagnosed types of head and neck tumors (Heroiu Cataloiu et al., 2013). Incidence rates of THCA had a significant upward trend in recent decades worldwide. It is estimated that the newly diagnosed cases of thyroid cancer are almost 50,000 in the United States in 2019 (Siegel et al., 2019). In China, the incidence rate of THCA in women has ranked fourth among the 10 most common cancers (Chen et al., 2016). Although patients diagnosed with THCA tended to have a favorable prognosis through surgery and radioiodine therapy, more than 10% of THCA patients will have a recurrence leading to considerable morbidity (Soares et al., 2014). Therefore, identifying reliable and accurate biomarkers for diagnosis and prognosis of THCA remains a challenge.

Long non-coding RNAs (lncRNAs) have been recognized as the major class of RNAs with more than 200 nucleotides that do not encode protein (Kopp and Mendell, 2018). A large body of literature has shown that lncRNAs play essential roles in a wide variety of biological processes, such as developmental and differentiation processes by lncRNA-mediated execution of gene expression programs (Fatica and Bozzoni, 2014; Marchese et al., 2017). High-throughput sequencing and profile analysis have identified a large number of differentially expressed lncRNAs in a multitude of cancers compared to normal tissues. In addition, aberrant lncRNA expression contributes to the development and maintenance of human cancers, thereby demonstrating the potential of lncRNAs as novel biomarkers in cancer diagnosis, prognosis, and as therapeutic targets (Spizzo et al., 2012; Sun et al., 2014; Huarte, 2015; Zhou et al., 2015a,b; Sanchez Calle et al., 2018; Zhou M. et al., 2018a,c; Bao et al., 2019). DNA methylation is one of the most common epigenetic mechanisms and is essential for the regulation of gene expression (Moore et al., 2013). It has been reported that epigenetic alterations in DNA methylation are also associated with various human cancers, including THCA (Stephen et al., 2011; Mancikova et al., 2014). Recent studies have reported aberrant DNA methylation of lncRNA promoters leading to perturbations of gene regulatory network, implying complex interplay between lncRNAs and DNA methylation (Zhao et al., 2016; Morlando and Fatica, 2018). However, crosstalk effects between DNA methylation and lncRNA regulation in THCA remain largely unknown.

In this study, we performed a genome-wide integrated analysis of methylation and the transcriptome to characterize the crosstalk between DNA methylation and lncRNA regulation, and identify epigenetically regulated lncRNAs. We further investigated the potential clinical value of these epigenetically regulated lncRNAs in a large number of THCA patients through bioinformatics analysis and experimental methods.

## Methods and Materials

### Patients and Tissue Samples

The clinical information of 507 THCA patients was downloaded from The Cancer Genome Atlas (TCGA, https://www.cancer.gov/) database.

Another in-house dataset including 119 PTC tissues and paired adjacent normal tissues were collected from patients who underwent surgery at the Second Affiliated Hospital of Harbin Medical University (HMU). No patients received any local or systemic treatments before the operation. The adjacent non-cancerous tissues were collected >2 cm from the tumor margins on the same or another lobe. All tissue samples were confirmed independently by two pathologists, blocked of formalin-fixed paraffin-embedded material and stored at 2–8°C with desiccation until use for later experiments. The clinicopathological characteristics of these patients are listed in Table 1. Informed consent was obtained for each patient, and the experiments were allowed by the Research Ethics Committee of HMU.

**Table 1:** The relation of *AC110011* expression and clinicopathological characteristics of PTC patients.

**Characteristics**	**Number**	**AC110011 expression**		***P*-value**
		**High**	**Low**	
**Gender**				
Male	13	7	6	0.7935
Female	106	53	53	
**Age (Years)**				
<45	67	30	37	0.1622
≥45	52	30	22	
**Multifocality**				
Yes	22	11	11	0.9652
No	97	49	48	
**Tumor size (cm)**				
≤2	98	44	54	0.0092^*^
>2	21	16	5	
**Lymph node metastasis**				
Yes	56	36	20	0.0043^*^
No	63	24	39	
**Extral thyroidal extension**				
Yes	23	19	4	0.0006^*^
No	96	41	55	
**TNM stage**				
I+II	106	51	55	0.1506
III+IV	13	9	4	

* *P ≤ 0.05 was considered significant*. *The bold values indicates that they have statistical significance*.

### Processing and Analysis of lncRNA Expression Profiles

RNA-seq data of 510 THCA tumor tissues and 58 non-cancer tissues based on the IlluminaHiSeq_RNASeq platform were retrieved from the UCSC Xena Browser (https://xena.ucsc.edu/). We annotate 15,873 lncRNAs from the RNA-seq data based on GENCODE (v23) annotations. Differential expression analysis was conducted using the R package “DESeq2.” Those lncRNAs with |log2(fold change)| >1 and False Discovery Rate (FDR)-corrected *p-*values < 0.05 were identified as differentially expressed lncRNAs. Hierarchical clustering analysis of samples based on lncRNA expression was performed using the R package “pheatmap” with the “ward.D2” method.

### Processing and Analysis of DNA Methylation Profiling

DNA methylation data of 515 THCA tumor tissues and 56 non-cancer tissues based on the Illumina Human Methylation 450 platform were retrieved from the UCSC Xena Browser (https://xena.ucsc.edu/). After removing probes with the missing value in more than 10% of samples, a total of 372,978 probes were stored for further analysis. Differentially methylated CpG loci between paired tumor tissues and non-cancer tissues were identified using a paired *t*-test with FDR-corrected *p-*values < 0.05 and absolute differences between group methylation mean (DGMB) > 0.04. Differentially methylated CpG loci between tumors with and without recurrence were determined using the R package “minfi” with FDR-corrected *p-*values < 0.05 and DGMB > 0.04.

### Survival Analysis

The random survival forest was carried out to identify the optimal combination of epigenetically regulated lncRNAs as novel signature for survival prediction through the R package “randomforestSRC” (Taylor, 2011). Kaplan-Meier survival curves and log-rank tests were used to compare survival differences between high-risk group and low-risk group with the R package “survival.” Univariate and multivariate Cox proportional hazards regression analyses were conducted through the R package “survival.” The time-dependent Receiver Operating Characteristic (ROC) curve was used to evaluate the performance of the signature for survival prediction using the R package “survivalROC.”

### Function Enrichment Analysis

Function enrichment analysis of Gene Ontology (GO) and Kyoto Encyclopedia of Genes and Genomes (KEGG) was carried out using the R package “clusterProfiler” (Yu et al., 2012). GO terms or KEGG pathways with adjusted *p*-value <0.05 were taken to be significantly enriched.

### RNAscope *in situ* Hybridization

Paraffin sections were disposed according to the agreement developed by the Advanced Cell Diagnostics. ISH was disposed using the RNAscope® 2.5 Assay (ACD, Inc. Catalog No. 322335) and RNAscope® 2.5HD Detection Kit -BROWN (ACD, Inc.Cat. No. 322310). RNAscope Probe-Hs-AC110011 and positive and negative control probes were ordered from ACD. These probes included positive control probes PPIB (positive control, Cat. No. 313901), and negative control probe DapB (negative control, Cat. No. 310043). The percentages of positive cancer cells were scored as follows: 0: none; 1: <10%; 2: 10–50%; and 3: >50%. A score of 2 was used to distinguish between low (<2) and high (≥2) levels of AC110011 gene expression. Images of the slides were analyzed using an OLYMPUS Dual-CCD microscope digital camera, and relevant semi-quantitative scores were acquired by estimating the punctate staining. In addition, half of the quantitative fraction was acquired by estimating the dot dyeing.

## Results

### Identification of Altered lncRNAs in the Development and Progression of THCA

**Figure 1:**
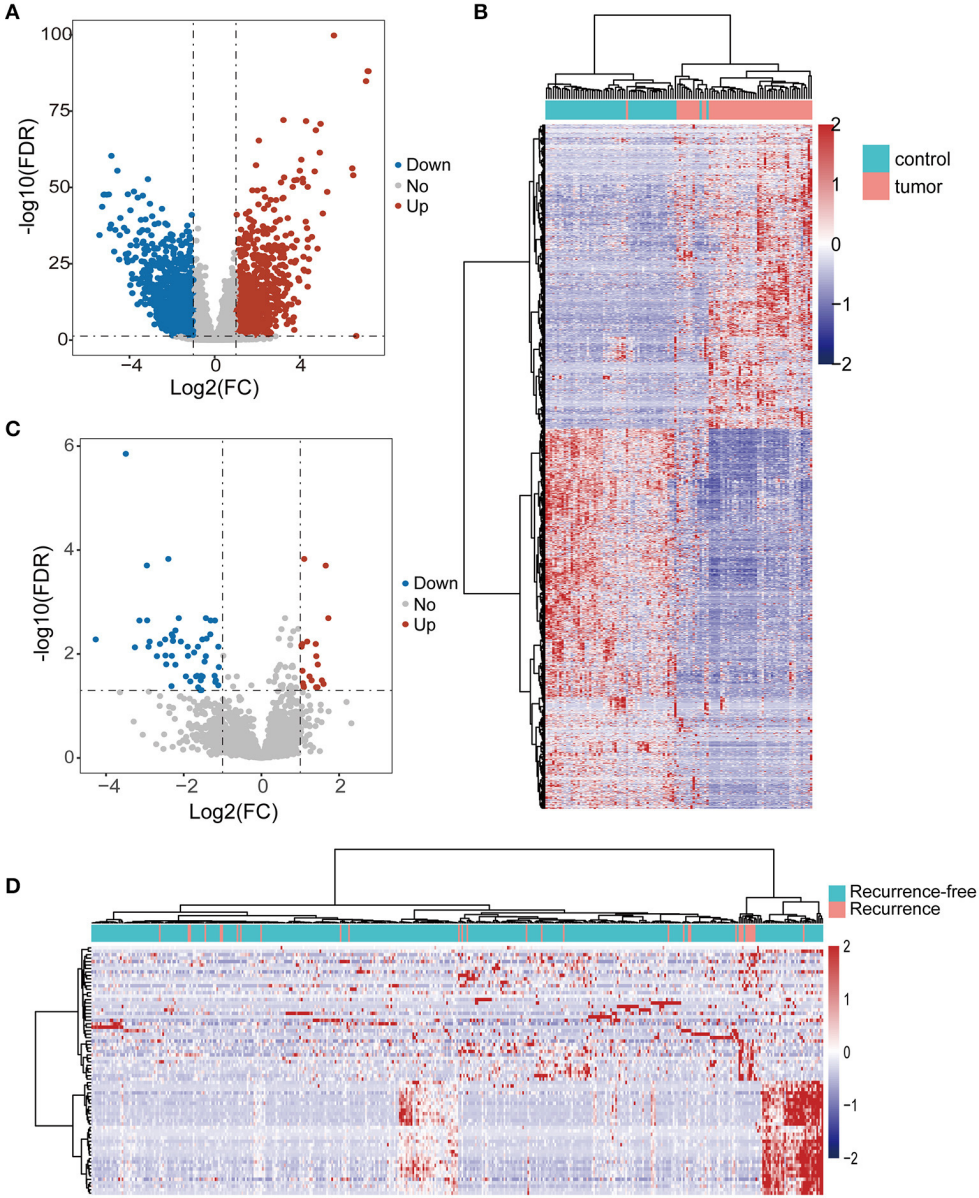
Identification of differentially expressed lncRNAs. **(A)** Volcano plot of differentially expressed lncRNAs between tumors and normal tissues. Blue dots indicate down-regulated lncRNAs and red dots denote up-regulated lncRNAs. **(B)** The hierarchical clustering heat map of tumor samples and non-cancer tissues based on differentially expressed lncRNAs. **(C)** Volcano plot of differentially expressed lncRNAs between tumors with and without recurrence. Blue dots indicate down-regulated lncRNAs and red dots denote up-regulated lncRNAs. **(D)** The hierarchical clustering heat map of tumor with and without recurrence based on differentially expressed lncRNAs.

We first performed differential expression analysis for lncRNAs in 58 pairs of tumors and non-cancer tissues of THCA. A total of 1,969 lncRNAs were found to be differentially expressed, including 868 up-regulated and 1,101 down-regulated lncRNAs in tumors compared to non-cancer tissues (Figure 1A). Hierarchical clustering analysis showed that the expression patterns of 1,969 lncRNAs were capable of distinguishing tumor samples from non-cancer tissues (Figure 1B). To identify lncRNAs associated with tumor recurrence, differential expression analysis for lncRNAs was undertaken between 32 recurrent tumors and 401 recurrence-free tumors. Finally, we identified 72 lncRNAs that were significantly altered in recurrent tumors vs. recurrence-free tumors. Of them, 21 lncRNAs were up-regulated and 51 lncRNAs were downregulated in recurrent tumors compared to recurrence-free tumors (Figure 1C). Hierarchical clustering analysis showed that all samples fell into two clusters that are significantly associated with recurrence status (*p* = 0.0008, Chi-square test; Figure 1D).

### Differential DNA Methylation Profiling During THCA Development and Progression

**Figure 2:**
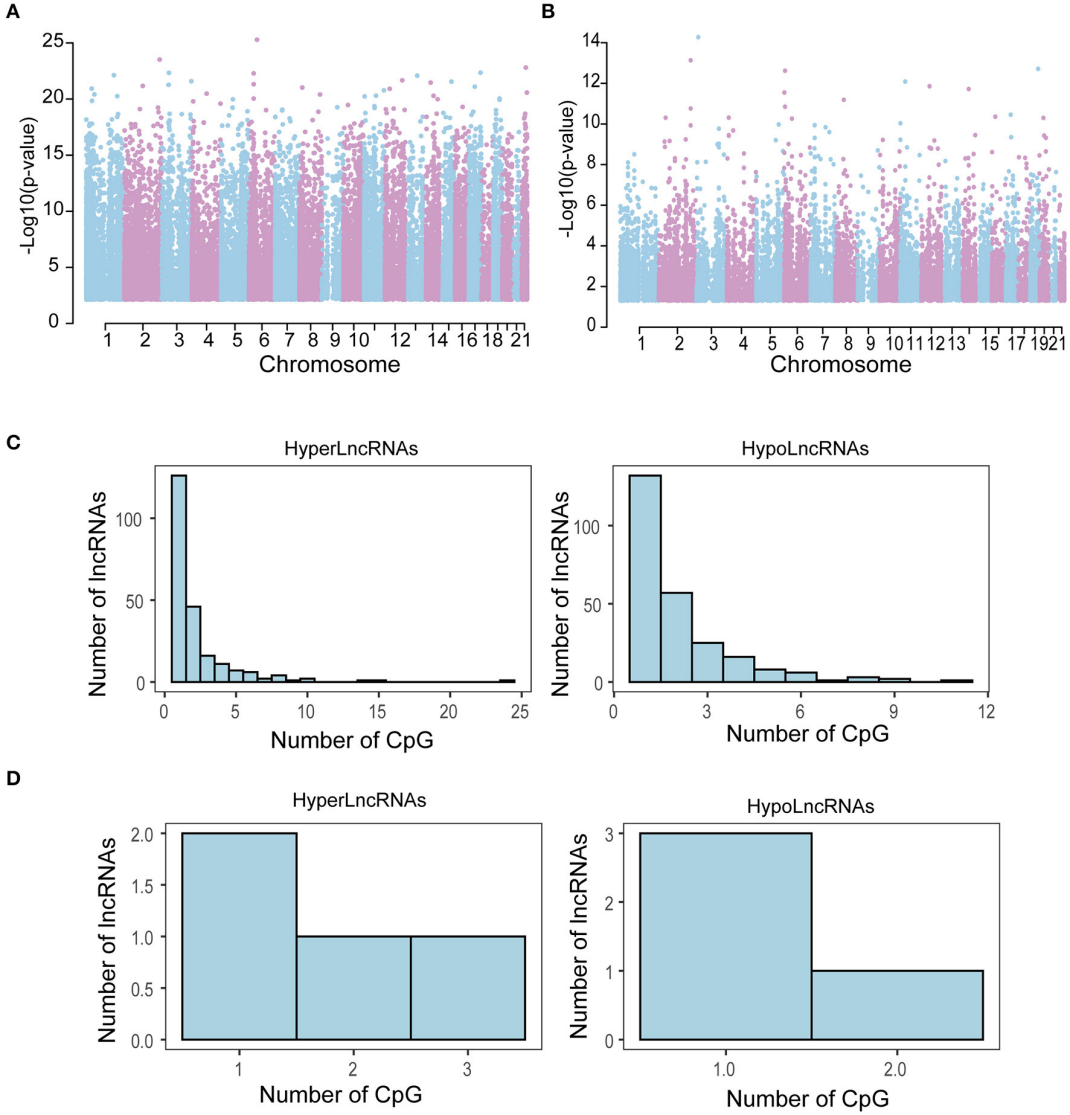
Chromosome distribution of differentially methylated CpG sites. A Manhattan plot was drawn for differentially methylated CpG sites between tumors and adjacent normal tissue **(A)** and between tumors with and without recurrence **(B)**. Distribution of HyperLncRNAs and HypoLncRNAs possessing different differentially methylated CpG sites associated with tumor development **(C)** and tumor recurrence **(D)**.

Further comparison of genome-wide DNA methylation on 56 pairs of tumor and adjacent normal tissues of THCA identified a total of 41,157 (11.03%) differentially methylated CpG sites, including 18,848 (45.8%) hypermethylated CpG sites (FDR < 0.05 and DGMB > 0.04) and 22,309 (54.2%) hypomethylated CpG sites (FDR < 0.05 and DGMB <-0.04) in tumors compared to adjacent normal tissue. To identify novel CpG islands aberrantly methylated in THCA recurrence, we also performed differential DNA methylation analysis between tumors with and without recurrence. We identified 20,283 (5.44%) differentially methylated CpG sites including 14,654 (72.25%) hypermethylated CpG sites (FDR < 0.05 and DGMB > 0.04) and 5,629 (27.75%) hypomethylated CpG sites (FDR < 0.05 and DGMB <-0.04) in tumors with recurrence compared to recurrence-free tumors. A Manhattan plot showed that these differentially methylated CpG sites are significantly associated with THCA development and progression is distributed across the methylome (Figures 2A,B).

### The Integrated Analysis Identifies Epigenetically Regulated lncRNAs in THCA

To identify epigenetically regulated lncRNAs (EpilncRNAs) in THCA, these differentially methylated CpG sites were mapped to 3 kb upstream and 3 kb downstream of differentially expressed lncRNAs. A total of 7,765 differentially methylated CpG sites were located in 689 up-regulated lncRNAs and 10,349 differentially methylated CpG sites were located in 873 down-regulated lncRNAs in tumors compared to adjacent normal tissues. For tumors with recurrence, there were 427 differentially methylated CpG sites located in 20 up-regulated lncRNAs and 334 differentially methylated CpG sites located in 40 down-regulated lncRNAs. Then we grouped these lncRNAs into categories based on DNA methylation in lncRNA: HyperLncRNAs and HypoLncRNAs. HyperLncRNAs are down-regulated lncRNAs with high DNA methylation levels, whereas HypoLncRNAs are up-regulated lncRNAs with low DNA methylation levels. Finally, we identified 475 methylation–driven lncRNAs (224 HyperLncRNAs and 251 HypoLncRNAs) associated with tumor development and 8 methylation-driven lncRNAs (4 HyperLncRNAs and 4 HypoLncRNAs) associated with tumor recurrence. These results demonstrated that methylation-mediated dysregulated lncRNA expression patterns involved in THCA development and progression. Furthermore, we found that 228 HyperLncRNAs displayed dissimilar DNA methylation patterns with 255 HypoLncRNAs (Figures 2C,D).

### Construction and Validation of an Epigenetically Regulated lncRNA-Based Prognostic Model (EpiLncPM) in THCA

**Figure 3:**
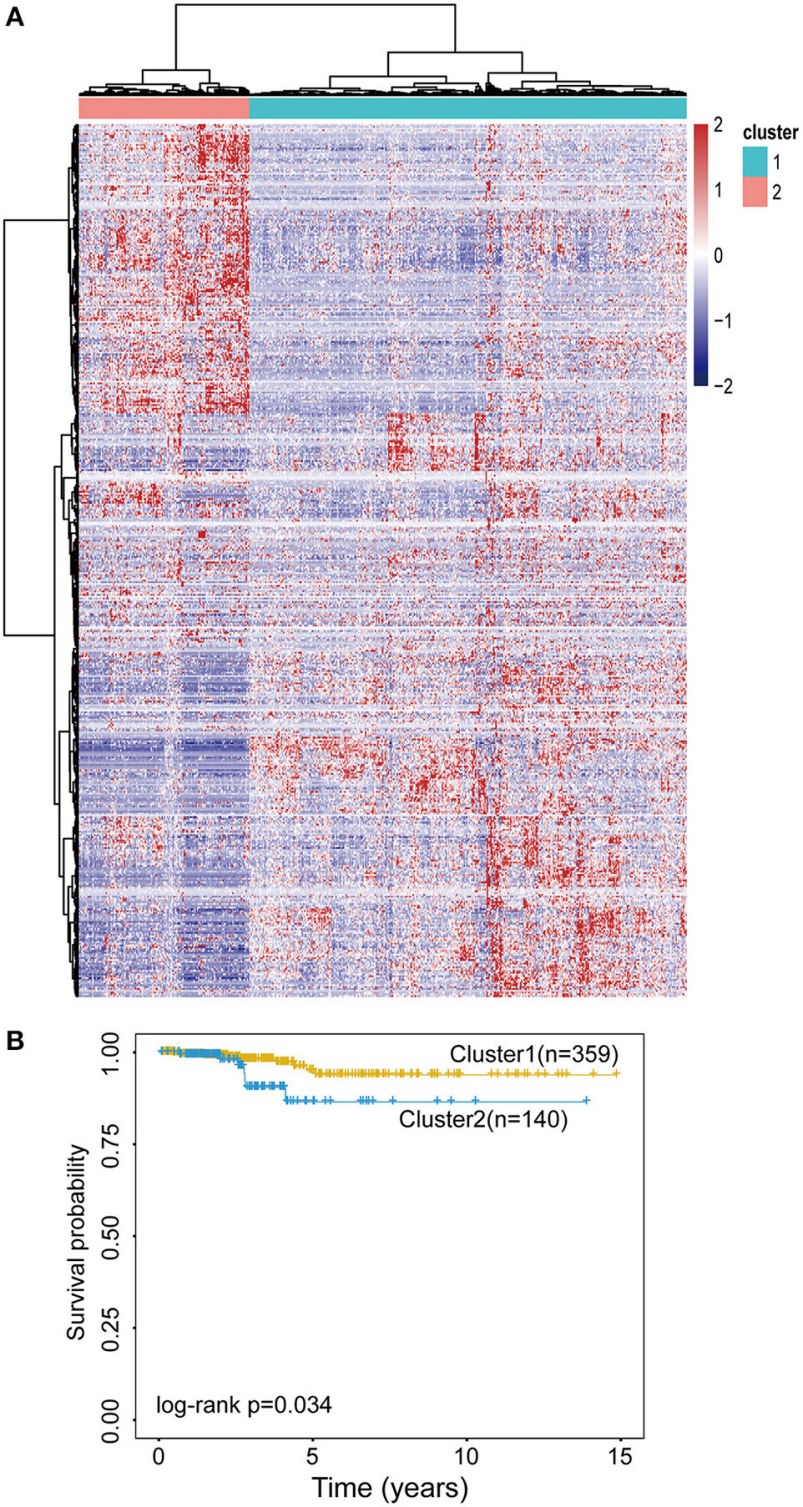
Evaluation of the prognostic value of the EpilncRNAs. **(A)** The hierarchical clustering heat map of THCA patients based on 483 EpilncRNAs. **(B)** Kaplan-Meier survival curves for two patient clusters.

**Figure 4:**
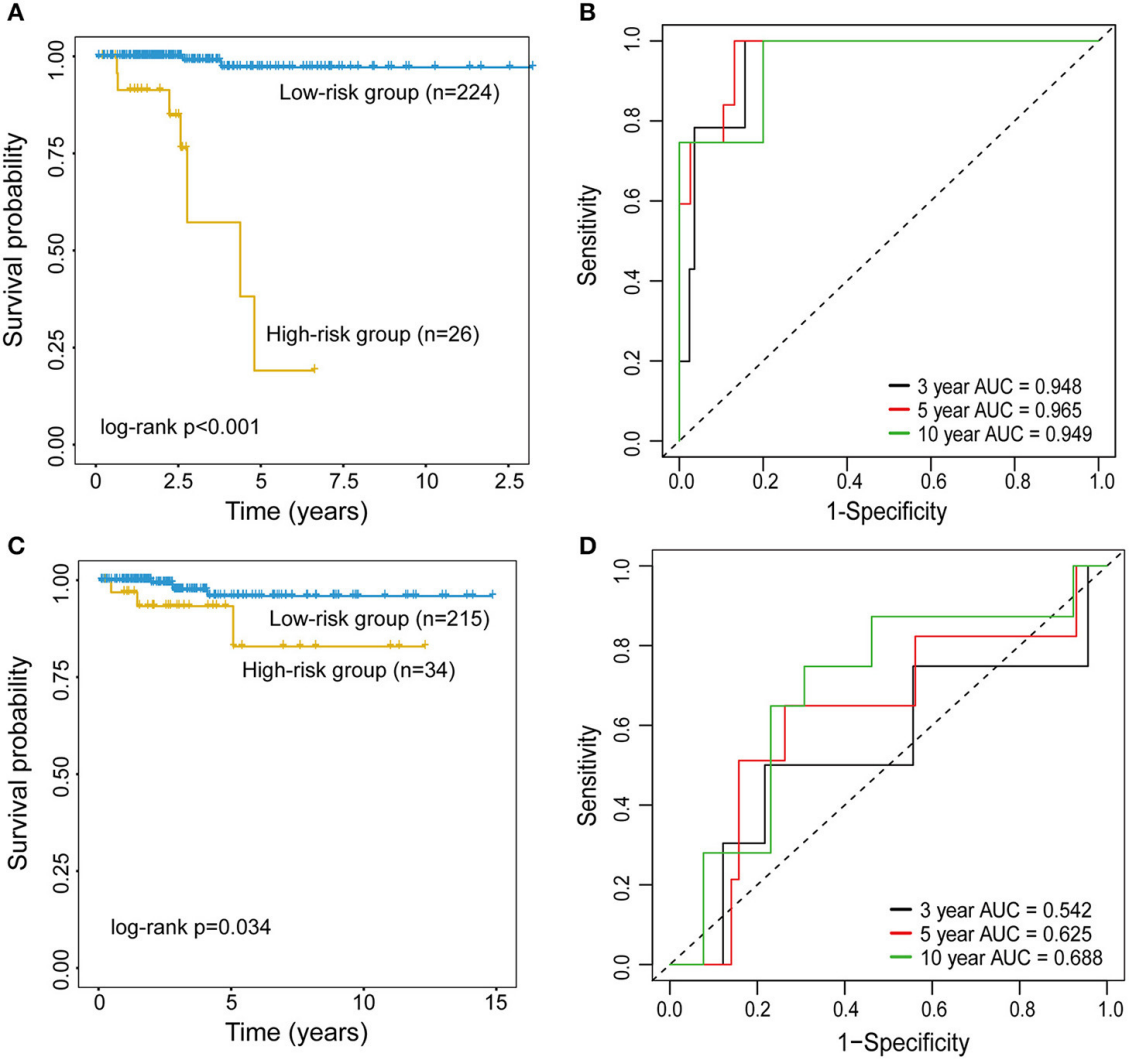
Performance evaluation of the EpiLncPM for survival prediction. Kaplan-Meier survival curves for the high- and low-risk groups in the training **(A)** and testing **(C)** datasets. The time-dependent receiver operating characteristic (ROC) curve analysis for survival prediction at three, 5 and 10-year survival rates in the training **(B)** and testing **(D)** datasets.

To examine whether epigenetically regulated lncRNAs have prognostic value for THCA patients, we first performed a hierarchical clustering analysis of all THCA patients based on the 483 methylation-driven lncRNAs. As shown in Figure 3A, all THCA were grouped into two patient clusters with significantly different survival (*p* = 0.034, log-rank test; Figure 3B), suggesting that epigenetically regulated lncRNAs may be used as biomarkers in survival prediction of THCA. Therefore, all THCA patients were randomly split into the training dataset (*n* = 250) and testing dataset (*n* = 249) and performed univariate Cox regression analysis for 475 methylation-driven lncRNAs in the training dataset. A total of 42 methylation-driven lncRNAs were significantly associated with survival. Thereafter, we performed the RSF on 42 candidate methylation-driven lncRNA biomarkers and identified an optimal combination of 5 methylation-driven lncRNAs as a prognostic signature. Finally an epigenetically regulated lncRNA-based prognostic scoring model (EpiLncPM) was developed in the training dataset using the expression levels of five optimal methylation-driven lncRNAs weighted by their coefficients from multivariate Cox regression analysis as follows: EpiLncPM = 13.1^*^ *AP006248.2*+2.53^*^AC068580.3+33.2^*^AC016396.2+3.12^*^LINC01140+1.19^*^LINC01135 (Table 2). Patients in the training dataset were divided into the high-risk group (n = 26) and low-risk group (n = 224) according to the optimal risk cutoff point from the R package “maxstat.” As expected, the survival analysis showed that patients with a low-risk score have a better prognosis than those with a high-risk score (*p* < 0.001, log-rank test; Figure 4A). The time-dependent ROC analysis showed that the AUC of the performance of the EpiLncPM for survival prediction at three, 5 and 10-year survival rates in the training dataset reached 0.948, 0.965, and 0.949, respectively (Figure 4B). When the EpiLncPM was applied to the testing dataset, patients in the high-risk group had significantly shorter survival than those in the low-risk group (*p* = 0.034, log-rank test; Figure 4C). The time-dependent ROC analysis showed that the AUC of the performance of the EpiLncPM for survival prediction at three, 5 and 10-year survival rates in the testing dataset reached 0.542, 0.625 and 0.688, respectively (Figure 4D).

**Table 2:** Detailed information of five optimal methylation–driven prognostic lncRNAs.

**Ensembl version**	**Ensembl name**	**Genomic location**	***P*-value**
ENSG00000279271.1	AP006248.2	Chromosome 8: 17,498,647-17,499,238(–)	0.008
ENSG00000235027.1	AC068580.3	Chromosome 11: 1,760,348-1,762,486 (+)	0.009
ENSG00000261076.1	AC016396.2	Chromosome 10: 58,325,614-58,327,030 (–)	0.002
ENSG00000267272.5	LINC01140	Chromosome 1: 87,129,765-87,169,198 (+)	0.023
ENSG00000234807.7	LINC01135	Chromosome 1: 58,785,128-58,901,109 (+)	0.030

### Independence of the EpiLncPM From Other Clinical Factors

Univariate Cox regression analysis showed that the EpiLncPM and stage are both significantly correlated with survival in the training and testing datasets (HR = 50.097, 95% CI 10.231-245.312, *p* < 0.001 for training dataset; HR = 4.395, 95% CI 0.981-19.686, *p* = 0.053; Table 3). Therefore, to investigate whether the EpiLncPM is an independent factor in predicting survival, we performed multivariate analysis including age, gender, stage, and the EpiLncPM. Results of multivariate analyses showed that the EpiLncPM still maintained a significant association with survival adjusting by other clinical factors (Table 3). These observations showed that EpiLncPM is an independent factor in predicting the survival of THCA patients.

**Table 3:** Univariate and multivariate analysis for survival in the training and testing datasets.

**Variables**		**Univariate analysis**			**Multivariate analysis**		
		**HR**	**95% CI**	***p*-value**	**HR**	**95% CI**	***p*-value**
**Training dataset (*****n*** **=** **250)**							
EpiLncPM	High vs. low	50.097	10.231–245.312	<0.001	29.050	5.94–142.074	<0.001
Age	Old vs. young	Inf	0-Inf	0.998	Inf	0-Inf	0.990
Gender	Female vs. male	1.307	0.271–6.305	0.739	0.237	0.247–6.509	0.776
Stage	III, IV vs. I, II	9.377	1.933–45.492	0.005	0.302	0.26–7.051	0.720
**Testing dataset (*****n=*** **249)**							
EpiLncPM	High vs. low	4.395	0.981–19.686	0.053	3.699	0.813–16.833	0.091
Age	Old vs. young	Inf	0-Inf	0.998	Inf	0-Inf	0.999
Gender	Female vs. male	0.202	0.045–0.915	0.038	0.268	0.058–1.233	0.091
Stage	III, IV vs. I, II	5.416	1.048–27.997	0.044	1.083	0.207–5.662	0.924

### Functional Implication of the EpiLncPM

**Figure 5:**
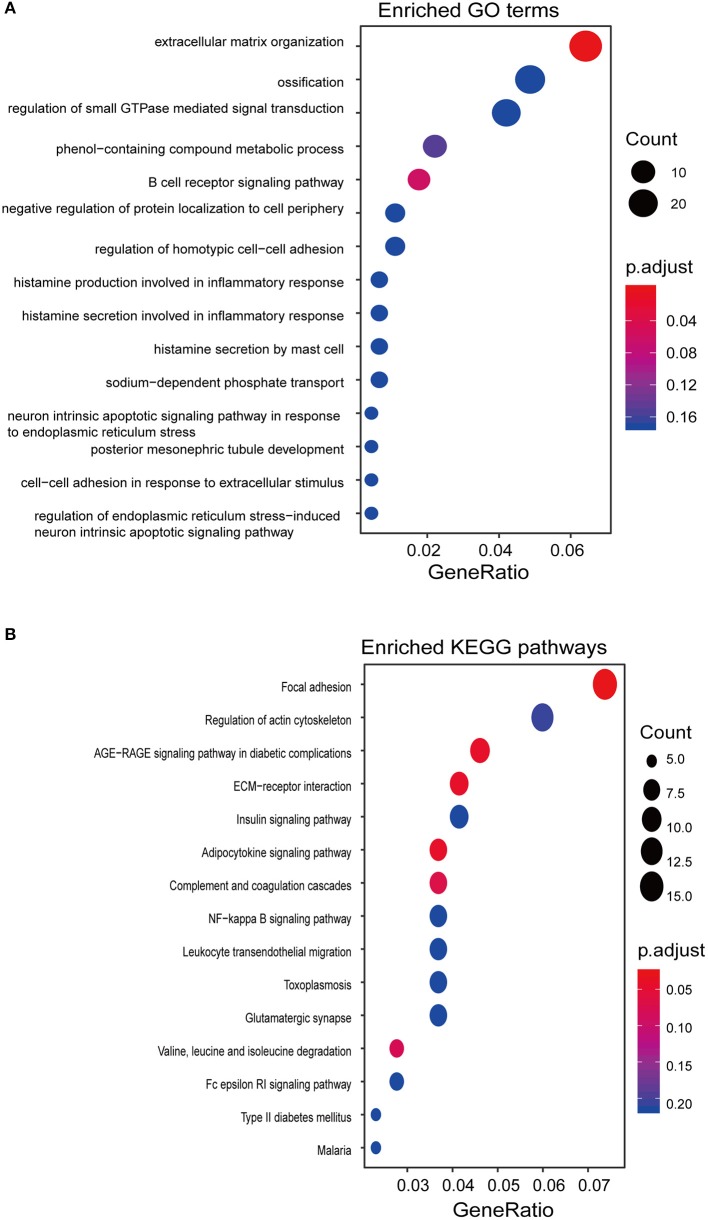
Functional prediction of the EpiLncPM. **(A)** Functional enrichment analysis of GO. **(B)** Functional enrichment analysis of KEGG.

To gain insights into the functional role of the EpiLncPM, we calculated the Pearson correlation coefficient between expression levels of lncRNAs and mRNAs using the EpiLncPM and identified 537 mRNAs that correlated with the EpiLncPM (Pearson correlation coefficient > 0.5). GO functional enrichment analysis revealed that the correlated mRNAs were significantly clustered in immune and inflammatory-related biological processes (Figure 5A). In addition, the KEGG enrichment analysis showed that the correlated mRNAs were enriched in known cancer-related pathways (Figure 5B).

### *In situ* Hybridization Analysis

**Figure 6:**
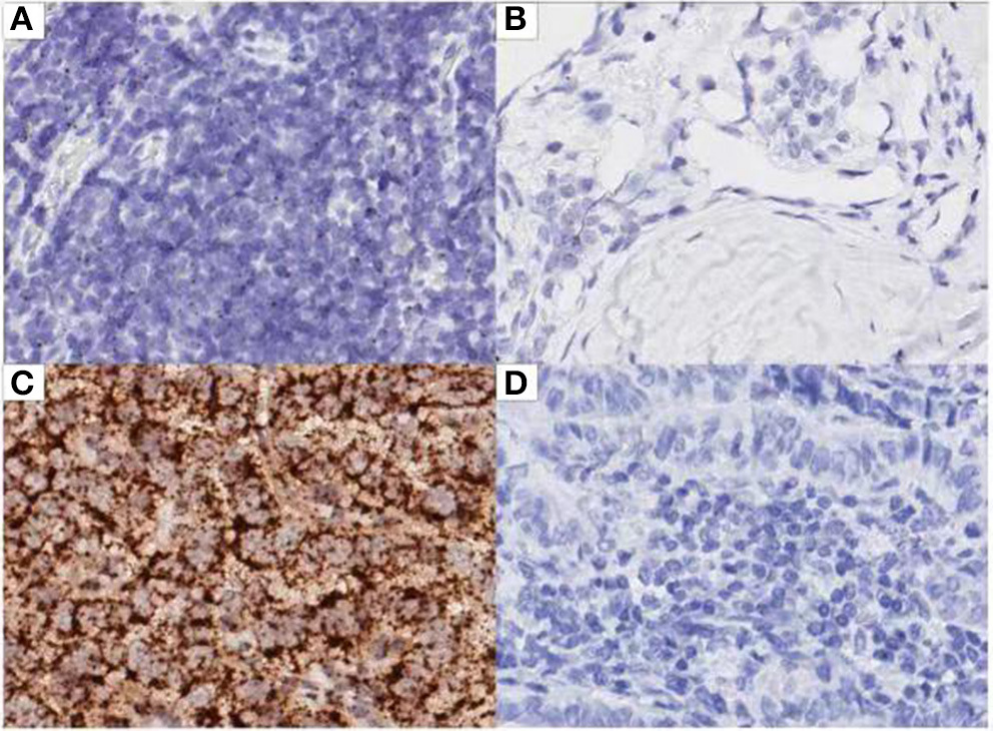
ISH analysis for lncRNA AC110011 in patient samples. **(A)** PTC tissue. **(B)** Adjacent non-neoplastic tissues. **(C)** Positive control. **(D)** Negative control.

The expression of AC110011 in 119 paraffin-embedded tissue samples of PTC and adjacent non-neoplastic tissues was semi-quantitatively examined by ISH. ISH showed that AC110011 was expressed in the nucleus (Figure 6). PTC tissues showed a significant increase in AC110011 expression as compared to that observed in adjacent non-neoplastic tissues. The AC110011 expression was statistically associated with tumor size (cm), lymph node metastasis, and extrathyroidal extension (Table 1).

## Discussion

Recent multi-omics analysis has demonstrated that THCA is a heterogeneous disease characterized by a high degree of molecular heterogeneity (Killock, 2014; Yoo et al., 2019), implying the potential of molecular profiling as a predictive factor in the diagnosis, prognosis and treatment response for THCA which will overcome the limitations of conventional clinical and histopathological features. mRNA and miRNA expression patterns have been widely investigated for a long time and many mRNA- or miRNA-based signatures have been identified to improve the diagnosis and prognosis of THCA (Santarpia et al., 2013; Han et al., 2018; Kim et al., 2018; Teng et al., 2018; Wang et al., 2019). In recent years, a new ncRNA class, lncRNA, has gradually become a research hotspot in diverse cancer fields. Numerous studies have demonstrated the superiority of lncRNA as diagnostic and predictive biomarkers in a diverse range of cancers compared to mRNAs and miRNAs (Zhou et al., 2015c, 2017, 2019; Zhou M. et al., 2018b). Aberrant lncRNA expression has also been observed in the development and progression of THCA (Yang et al., 2016; Lu et al., 2018), and several lncRNA biomarkers have been identified for THCA diagnosis and prognosis (Li et al., 2017; Liu et al., 2018; Liang and Sun, 2019).

Emerging evidence has shown that DNA methylation is an important epigenetic regulator of lncRNA expression and epigenetic changes in DNA methylation can disturb the expression pattern of lncRNAs and contributes to carcinogenesis (Heilmann et al., 2017; Tang, 2018; Zhou Z. et al., 2018; Bao et al., 2019). However, the interplay between lncRNA regulation and DNA methylation in the development and progression of THCA is still largely unknown. In this study, we performed an integrated analysis of methylation and the transcriptome to characterize the dysregulated DNA methylation pattern of lncRNAs. By performing a differential expression analysis, we observed genome-wide changes in lncRNA expression and DNA methylation during THCA development and progression. By directly mapping altered DNA methylation to the flaking regions of lncRNAs, we found that a total of 483 differentially expressed lncRNAs were epigenetically deregulated, which were defined as methylation-driven lncRNAs. These lncRNAs can be separated into two categories based on their methylation patterns and expression levels, between tumor and normal samples or between tumors with and without recurrence.

Despite the fact that several previous studies have identified a host of lncRNAs with prognostic roles in THCA, knowledge about the clinical value of methylation-driven lncRNAs remains limited. We performed clustering analysis for THCA patients based on the expression patterns of methylation-driven lncRNAs, which were found to be able to distinguish patients with different prognoses, thereby demonstrating a potential function in the prognosis of THCA. Therefore, we identified a methylation-driven 5-lncRNA-based signature (EpiLncPM) to improve the prognosis prediction utilizing the RSF and multivariate Cox analysis. This EpiLncPM was validated by the training and testing datasets. Moreover, the prognostic capacity of the EpiLncPM is independent of other clinical and pathological factors for the survival of patients with THCA. By performing function enrichment analysis for mRNAs co-expressed with the EpiLncPM, we gained insight into the potential functional relevance of methylation-driven lncRNAs in THCA. We found that the EpiLncPM is involved in immune and inflammatory-related biological processes, such as B cell receptor signaling pathways, histamine production involved in inflammatory responses, histamine secretion by mast cells, and known cancer-related pathways. It has been reported that chronic inflammation and the tumor microenvironment play critical roles in cancer development and progression, including THCA (Cunha et al., 2014; Rotondi et al., 2018; Ferrari et al., 2019). For example, mast cell infiltrates are linked to extrathyroidal extension and invasiveness (Visciano et al., 2015). NF-κB is associated with inflammatory and immune responses and its increased activity correlate with a more aggressive phenotype of THCA (Giuliani et al., 2018). Finally, we validated the functional roles of one selected methylation-driven lncRNA signature by *in situ* hybridization analysis in 119 PTC tissues and paired with adjacent normal tissues.

In summary, we performed a genome-wide integrated analysis of methylation and the transcriptome to characterize the crosstalk between DNA methylation and lncRNA, and identify epigenetically regulated lncRNAs. Additionally, we identified a methylation-driven 5-lncRNA-based signature (EpiLncPM) with potential clinical application in predicting the prognosis of THCA.

## Data Availability Statement

Clinical information of THCA patients was downloaded from The Cancer Genome Atlas (TCGA, https://www.cancer.gov/) database. RNA-seq data and DNA methylation data of tumor tissues and non-cancer tissues were retrieved from the UCSC Xena Browser (https://xena.ucsc.edu/).

## Ethics Statement

The studies involving human participants were reviewed and approved by the Research Ethics Committee of Harbin medical university. The patients/participants provided their written informed consent to participate in this study.

## Author Contributions

CW and YS conceived and designed the experiments. QL, PW, and CS performed the experiments and analyzed the data. QL, CW, and YS wrote the paper. All authors read and approved the final manuscript.

### Conflict of Interest

The authors declare that the research was conducted in the absence of any commercial or financial relationships that could be construed as a potential conflict of interest.
